# Pulmonary fibrosis: Is stem cell therapy the way forward?

**DOI:** 10.1016/j.jtumed.2023.09.009

**Published:** 2023-10-11

**Authors:** Muhammad Ikrama, Muhammad Usama, Shifa Israr, Maryam Humayon

**Affiliations:** Services Institute of Medical Sciences, Department of Medicine, Lahore, Pakistan

**Keywords:** أمراض الرئة الشعاعية, أمراض الرئة, التليف الرئوي, الطب التجددي, الخلايا الجذعية, Interstitial lung diseases, Lung diseases, Pulmonary fibrosis, Regenerative medicine, Stem cells

## Abstract

Pulmonary fibrosis, a chronic and fatal lung disease affecting millions of people worldwide, is characterized by the scarring of lung tissue, thereby impairing oxygen exchange between the lungs and blood. The etiology of pulmonary fibrosis is multifactorial, involving environmental exposures, comorbidities, and genetic mutations. Current pharmacological treatments can only slow the disease progression, and lung transplantation is limited by donor availability and complications. Stem cell therapy has emerged as a potential alternative treatment for pulmonary fibrosis, in which stem cells modulate the inflammatory response, differentiate into lung epithelial cells, secrete growth factors and extracellular matrix components, and enhance vascularization and tissue regeneration. Various sources of stem cells, such as endogenous lung stem cells, embryonic stem cells, induced pluripotent stem cells, and mesenchymal stem cells, have been investigated in animal models and human trials. Various delivery routes, such as intravenous injection, intratracheal instillation, and inhalation, have been tested for safety and efficacy. However, several challenges and limitations remain to be overcome, such as high costs, ethical issues, immunological compatibility, cell survival and homing, and long-term outcomes. Further research is needed to optimize the protocols and parameters in stem cell therapy for pulmonary fibrosis, and to evaluate the clinical benefits and risks for patients.

## الملخص

التليف الرئوي مرض رئوي مزمن وقاتل يؤثر في ملايين الأشخاص في جميع أنحاء العالم. يتميز بتندب النسيج الرئوي، مما يعيق تبادل الأكسجين بين الرئتين والدم. إن أسباب التليف الرئوي متعددة العوامل، وتتضمن التعرض للبيئة والأمراض المصاحبة والطفرات الجينية. يمكن للعلاجات الدوائية الحالية فقط أن تبطئ من تقدم المرض، في حين أن زراعة الرئة محدودة بسبب توفر الأعضاء والمضاعفات. وقد ظهر العلاج بالخلايا الجذعية كبديل محتمل لعلاج التليف الرئوي. تتمتع الخلايا الجذعية بالقدرة على تعديل الاستجابة المناعية، وتحول إلى خلايا بطانية رئوية، وإفراز العوامل النموذجية ومكونات المصفوفة الخارجية للخلية، وتعزيز التوصيل الدموي وتجديد الأنسجة. تمت دراسة مصادر مختلفة من الخلايا الجذعية، مثل الخلايا الجذعية الرئوية الداخلية، والخلايا الجذعية الجنينية، والخلايا الجذعية متعددة القدرات المستحثة، والخلايا الجذعية اللحمية، في نماذج حيوانية وتجارب بشرية. تم اختبار طرق التوصيل المختلفة، مثل الحقن الوريدي، والتثبيت عبر القصبة الهوائية، والاستنشاق، لأمانها وفعاليتها. ومع ذلك، لا يزال هناك العديد من التحديات والقيود التي يجب التغلب عليها، مثل التكاليف العالية، والقضايا الأخلاقية، والتوافق المناعي، وبقاء الخلايا والتوجيه، والنتائج على المدى الطويل. هناك حاجة إلى مزيد من البحث لتحسين البروتوكولات والمعايير للعلاج بالخلايا الجذعية للتليف الرئوي ولتقييم فوائده ومخاطره السريرية للمرضى.

## Introduction

Pulmonary fibrosis (PF), a member of the enigmatic interstitial lung disease family, involves progressive scarring of lung tissue. In PF, the interstitium, a delicate network supporting the lung's air sacs, succumbs to the invasion of fibrous scar tissue. Consequently, the lungs become stiff and incapable of efficient expansion and contraction, and oxygen exchange is ultimately compromised. PF is not one disease, but many. Some people may live with PF for years, whereas others may die within months.^1^

Although the origins of PF remain elusive in some cases, several factors contribute to its emergence: (1) long durations of exposure to harmful substances in the environment or the workplace, such as dust from silica, asbestos, coal, grains, or excrement from birds or animals; (2) certain medical conditions affecting the lungs or immune system, such as rheumatoid arthritis, scleroderma, sarcoidosis, and tuberculosis; (3) radiation therapy for lung or breast cancer that harms healthy lung tissue; (4) use of certain medications that can cause inflammation or damage to the lungs, such as chemotherapy drugs, antibiotics, and anti-inflammatory drugs; (5) genetic factors predisposing people to PF; and (6) infections causing direct lung damage and abnormal healing. In most cases, PF has no known cause and is termed as idiopathic PF.^2^^,^^3^

The above factors induce recurrent or chronic damage to the alveolar epithelial cells (AECs), which cover the air sacs in the lungs. AECs have a crucial role in maintaining lung balance and repair.

**Figure 1:**
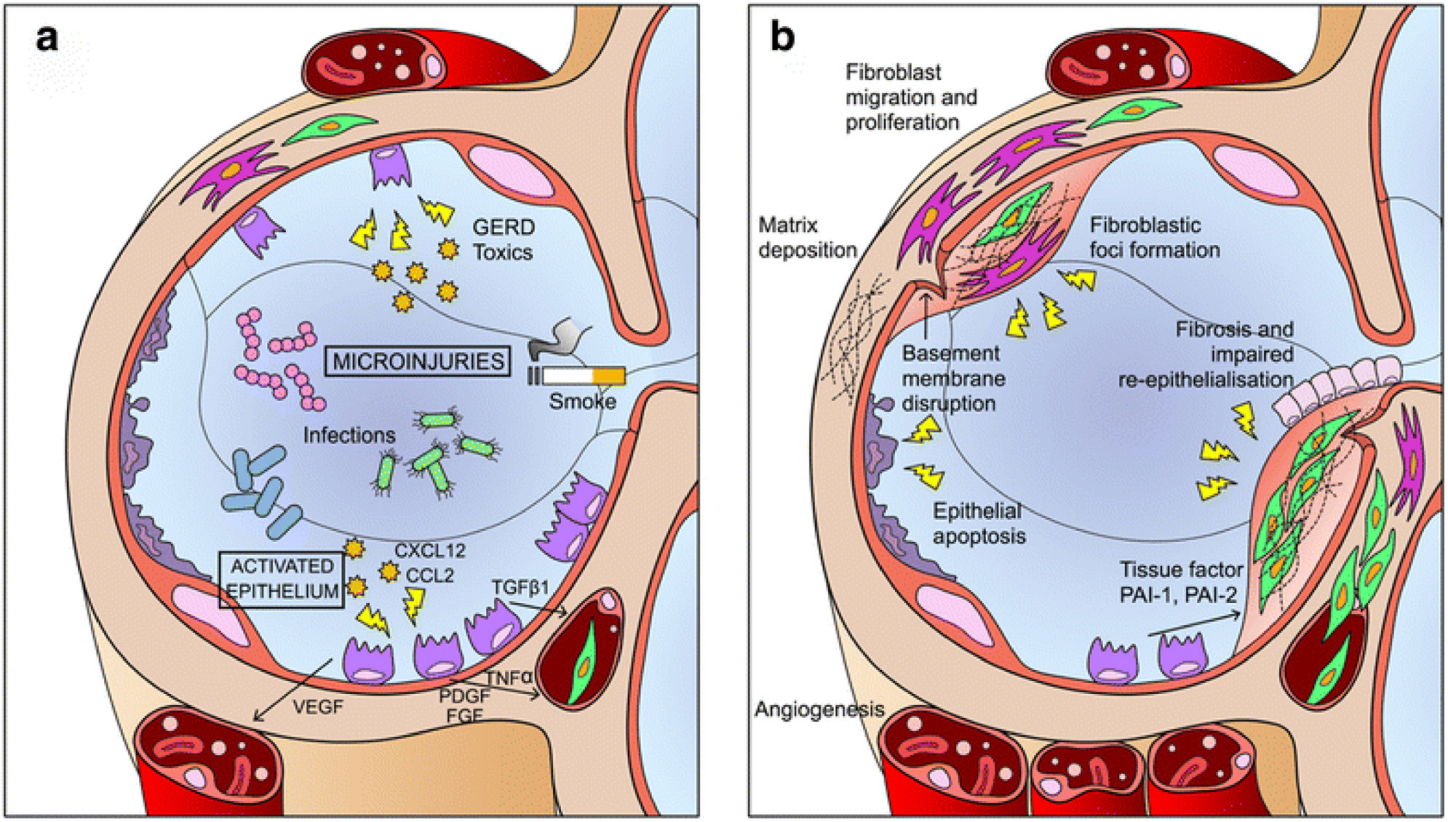
Adapted from Sgalla et al.^5^ Schematic view of IPF pathogenesis. Repeated injuries over time lead to maladaptive repair processes, characterized by alveolar epithelial type II (AEC2) apoptosis, proliferation and epithelial-mesenchymal cross-talk (a), and proliferation of fibroblasts and myofibroblasts, and accumulation of extracellular matrix (b). CCL2, chemokine C-C motif ligand 2; CXCL12, C-X-C motif chemokine 12; FGF, fibroblast growth factor; PAI-1, plasminogen activator inhibitor 1; PAI-2, plasminogen activator inhibitor 2; PDGF, platelet-derived growth factor; TGF-β1, transforming growth factor-beta 1; TNF-α, tumor necrosis factor-alpha; VEGF, vascular endothelial growth factor.

Apoptosis of AECs leads to a loss of epithelial structure and function. Consequently, the release of pro-inflammatory and pro-fibrotic mediators—such as cytokines and transforming growth factor-beta (TGF-β), platelet-derived growth factor (PDGF), and fibroblast growth factor (FGF), which stimulate fibroblast growth—results in excessive and persistent extracellular matrix deposition and remodeling,^4^ as shown in Figure 1.^5^

Because of its insidious nature, PF has an inconspicuous onset of symptoms. An unyielding dry cough and subtle breathlessness during physical activity are often the initial warning signs. As the disease progresses, fatigue, chest discomfort, unexplained weight loss, and finger clubbing become apparent.

The elusive nature of PF necessitates a comprehensive diagnostic approach. A thorough evaluation of medical history and a meticulous physical examination provide the first diagnostic clues. Lung function tests and imaging studies, including chest X-rays and computed tomography scans, offer invaluable insights into the extent of fibrosis. In certain cases, a lung biopsy may be essential to confirm the diagnosis and rule out other conditions.^6^

Although no cure for PF exists, treatments include oxygen therapy, medications, pulmonary rehabilitation and lung transplantation, among which lung transplantation appears to have the highest likelihood of curing the disease.^7^ This review is aimed at describing the current status of stem cell therapies for PF.

## Treatment modalities

The treatment options available for PF are detailed as follows and shown in Table 1.^8^

**Table 1:** Treatment options for IPF. From Glass et al.^8^

Treatment	Mechanism of action	Clinical effects
**Current therapies**		
Pirfenidone	Antifibrotic and anti-inflammatory	Slows rate of decline in FVC
Nintedanib	Antifibrotic and anti-inflammatory	Slows rate of decline in FVC
Oral corticosteroids, opioids	Antitussive	Decreases cough and improves quality of life
Anti-acids, proton pump inhibitors	Decreases GERD	Benefits unclear
Lung transplantation	Surgical replacement of one or both lungs	Available only as a potentially curative therapy
**Therapies in development**		
PRM-151	Recombinant human pentraxin-2; acts as an antifibrotic agent	Slows rate of decline in FVC
Pamrevlumab	Fully human recombinant monoclonal antibody to CTGF	Slows rate of decline in FVC
TD139	Small molecule inhibitor of galectin-3	Decreases plasma biomarkers of inflammation; study in progress to assess effect on FVC
PLN-74809	Blocks activation of the TGFβ pathway	Study in progress with end-points of safety, tolerability, and pharmacokinetics
TRK-250	Suppresses TGFβ expression	Study in progress to assess the safety and tolerability of single and multiple inhaled doses

Abbreviations: CTGF, connective tissue growth factor; FVC, forced vital capacity; GERD, gastroesophageal reflux disease; IPF, idiopathic PF.

### Medications

Among biologic agents, pirfenidone and nintedanib are two commonly used drugs that are FDA approved. Nintedanib inhibits the production and secretion of multiple types of collagen and fibronectin, and affects the function of two proteins that help collagen I fold and assemble. Consequently, fibrotic tissue formation and scarring in the lungs decrease. Pirfenidone achieves similar effects by blocking TGF-β, a molecule that stimulates collagen synthesis. These biologic agents are being used for cases of idiopathic PF (IPF) of any severity. Other types of PF (not IPF) may benefit from treatment with steroids or drugs that decrease inflammation.^9^, ^10^, ^11^

### Oxygen therapy

This treatment uses a device that supplies oxygen through a nasal tube or a face mask. It has been demonstrated to have potential benefit for patients with PF.^12^

### Pulmonary rehabilitation

This treatment helps improve physical function in patients with PF by providing exercise training, education and counseling, thereby increasing quality of life; decreasing dyspnea, and increasing exercise capacity.^13^

### Lung transplantation

This surgery involves replacing one or both damaged lungs with healthy lungs from a donor. Lung transplantation is usually an option for patients with advanced PF who have not benefitted from other treatments. This procedure poses substantial risks such as infection, rejection and problems associated with the use of immunosuppressive drugs.^7^

Smoking cessation, environmental allergen avoidance and regular vaccinations, such as influenza and pneumonia vaccination, are recommended to decrease the risk of respiratory infections and consequently slow the progression of PF.^14^^,^^15^

Researchers are attempting to find new ways to diagnose, treat, and prevent PF. Some examples of ongoing research include the following:

• *Creating new biomarkers* (biological signs) to identify people at risk of developing PF or to track their disease status^5^
• *Evaluating new medications* targeting specific pathways involved in lung scarring or inflammation
• *Investigating gene therapy* or *stem cell therapy* as possible methods of repairing damaged lung tissue or preventing further scarring^16^
• *Modifying surgical techniques* and outcomes for lung transplantation as a last resort for patients with untreatable PF^17^

## Stem cells: a new era

Stem cell therapy, a potentially promising option for PF treatment, uses pluripotent stem cells derived from a variety of sources including mesenchyme, embryos, or endogenous lung stem cells. Some of the evidence-based treatment effects include the following.

### Regulating inflammation

Stem cells exert immunomodulatory effects and attenuate inflammation in the lungs. These processes are essential for inhibiting the progression and exacerbation of lung fibrosis.

### Repairing lung tissue

Stem cells secrete anti-fibrotic and angiogenic factors that promote healing of the damaged tissue.

### Restoring organ function

Stem cells replace dysfunctional cells by differentiating into different types of cells, thus reversing the disease process.

### Bioartificial organs

Another promising avenue is the engineering of bioartificial organs by using either natural or synthetic frameworks to generate functional lung tissue for medical use. For production of natural organ scaffolds, native tissues are subjected to decellularization, leaving behind acellular structures that retain the original architecture of the organ. These structures are then seeded with the patient's own cells to encourage the regeneration of functional tissue. Various techniques for decellularization have been used for lung tissue, although consensus is lacking regarding the most effective approach.^18^

Stem cell therapy for PF remains under investigation, and further clinical trials are needed to confirm its safety and efficacy. However, this treatment offers hope for patients with this debilitating disease.^19^

## Types of stem cells

The following types of stem cells can be used to treat PF.

### Endogenous lung stem/progenitor cells

These cells reside in the lung tissue and can regenerate damaged alveoli and bronchioles. However, their number and function may be impaired in PF, owing to chronic inflammation and fibrosis.^20^

### Embryonic stem cells (ESCs)

These cells are isolated from blastocysts, and may have the potential to differentiate into cells from any of the three germ layers, by virtue of being pluripotent. However, their application is restricted by ethical concerns, immunological incompatibility, and tumorigenicity.^21^

### Induced pluripotent stem cells (iPSCs)

These body cells have reverted to a state in which they can be induced to become any type of cell through the addition of specific transcription factors.^22^ They have similar characteristics to ESCs, but avoid ethical and immunological problems. However, they pose a risk of tumorigenicity and may retain epigenetic memory of their original cell type.

### Mesenchymal stem cells (MSCs)

These stem cells can be extracted from sources such as fat cells, blood from the umbilical cord, bone marrow, or placenta. They can transform into mesodermal lineages, such as chondrocytes, osteocytes, or adipocytes, etc., and can differentiate into ectodermal and endodermal cells under specific conditions. They also have immunomodulatory properties and secrete various growth factors, cytokines, chemokines, and extracellular vesicles, which modulate inflammation and fibrosis. This cell type has shown the greatest potential in experimental studies and thus is the focus of this review.^23^

## Routes of administration

The delivery methods for stem cell therapy in patients with PF include the following.

### Intravenous injection

This route, the most frequently used for administration for MSCs, enables systemic distribution of MSCs throughout the body via the blood circulation.^24^ However, it also poses challenges, such as low engraftment efficiency in the lungs due to pulmonary capillary filtration or clearance by other organs.^25^

### Intratracheal instillation

This direct delivery method enables lung-specific targeting of stem cells. It involves injecting MSCs or other types of stem cells into the trachea through a syringe or catheter. This method has advantages such as high local concentration of stem cells in the lungs.^26^

### Inhalation

This direct delivery method for lung-specific targeting of stem cells involves nebulizing stem cells into aerosols that are inhaled by patients via a mask or mouthpiece. Inhalation can avoid several problems associated with intravenous injection, such as cell trapping in the lungs, poor extravasation, and thrombus formation; however, it requires more complex formulation and delivery methods.^25^

## Mesenchymal stem cells: current status

According to a recent meta-analysis of 24 preclinical studies on MSC therapy for PF, MSC therapy trials have resulted in an increased survival rate from PF compared to the control groups. However, these studies had high heterogeneity and risk of bias, and the optimal dose, timing, source, and delivery route of MSCs were not well established.^27^

According to another review and ClinicalTrials.gov, few studies have investigated stem cell therapy for PF, either clinically or preclinically, and most have involved small sample sizes, short follow-up periods, inconsistent outcome measures, and a lack of placebo control.^28^ Several studies are discussed below.

A phase I clinical trial using allogeneic human mesenchymal stem cells (hMSCs) in patients with PF has assessed the safety of the intravenous administration of bone marrow hMSCs from young, unrelated men to nine patients with mild to moderate IPF. The patients received a single intravenous infusion of 20, 100, or 200 million hMSCs per infusion. No treatment emergent serious adverse events were observed at week 4 postinfusion in all patients. The trial also reported 21 adverse events in seven patients, none of which were associated with the treatment. Two patients in the highest dose cohort died before study completion, because of disease severity. The secondary end-points showed variable changes in lung function, 6-min walk test (6MWT), and quality of life over the 60-week study period; however, the data were exploratory, and the study was not powered for statistical significance.^29^

Another phase I trial assessing the associated risks and the possibility of use of MSCs derived from bone marrow MSCs endobronchially, in patients with mild or moderately severe IPF, has indicated that the treatment had a low risk profile, but did not alter the course of disease progression. Some patients had adverse events such as fever, dyspnea, cancer, or death, but these outcomes were not likely to have been related to the treatment. Moreover, some bone marrow MSCs had chromosomal abnormalities, thus potentially limiting the use of cells sourced from patients with IPF themselves.^30^

In a pilot study assessing the efficacy of intratracheal instillation of bone marrow-derived cells in patients with silicosis, a fibrotic lung disease caused by silica exposure, five patients receiving cell therapy were followed for 1 year. No serious adverse effects were observed, and some signs indicated improved lung perfusion. The authors have suggested that this approach could be further investigated in larger trials.^31^

A case report has described that a severely ill patient with COVID-19 and PF receiving mechanical ventilation showed improvements after receiving two doses of umbilical cord-derived MSCs. The patient showed increased oxygenation, decreased inflammation, improvements in chest computed tomography imaging findings, and modulation of immune cell populations and cytokines after the cell therapy. No serious adverse events of the intervention were noted. The findings suggest that MSCs may be a promising intervention for such patients.^32^

A study has indicated that human adipose-derived MSCs (AD-MSCs) significantly increase survival and decrease fibrosis in mice with bleomycin-induced PF. The AD-MSCs were found to engraft into the damaged lung tissue and exert anti-inflammatory as well as anti-fibrotic effects through the mechanisms already described. AD-MSCs also achieved better preservation of lung architecture and function than pirfenidone, a standard drug for IPF. The authors suggest that AD-MSCs may be a promising choice for patients with PF in initial disease stages.^33^

These results suggest that stem cell therapy may have beneficial effects on pulmonary function and quality of life in patients with PF. However, more rigorous and larger-scale clinical trials are needed to confirm the benefits and risks of stem cell therapy for PF.

## Optimizing stem cell therapy for PF

Optimizing stem cell therapy for PF is a challenging but important task to improve the therapeutic outcomes and safety of this novel approach. Some uncertainties and challenges that must be addressed include the following.

### Exploring different cell sources

Although MSCs are the most widely used type of stem cells for PF, other types of stem cells may have advantages in terms of differentiation potential, immunogenicity, availability, and ethical issues. For example, iPSCs can be derived from patients' somatic cells and differentiated into lung-specific cell types, such as ATII cells or lung spheroids. However, iPSCs also have drawbacks, such as low efficiency of reprogramming; risk of genomic instability and tumorigenicity; and technical difficulties in large-scale production.^34^ Therefore, more studies are needed to compare the efficacy of different cell sources for PF treatment.

### Refining delivery methods and dosing regimens

The optimal route, frequency, timing, and dose of stem cell administration for PF remain unclear. Different delivery methods may have varying effects on the biodistribution, retention, and homing of stem cells in the lungs. For example, intravenous injection may enable systemic distribution, but may also result in low engraftment efficiency due to pulmonary capillary filtration or clearance by other organs. Intratracheal instillation or inhalation may achieve higher local concentrations, but may also cause airway obstruction or inflammation.^16^, ^17^, ^18^ Therefore, more studies are needed to optimize the delivery methods and dosing regimens of stem cell therapy for PF, to enhance the therapeutic benefits, and avoid potential harmful effects.

### Investigating the underlying mechanisms of action

The exact mechanisms through which stem cells exert therapeutic effects on PF remain poorly understood. Stem cells are generally believed to modulate inflammation and fibrosis through their paracrine actions rather than direct differentiation into lung tissue.^6^ However, the specific factors involved in this process, such as growth factors, cytokines, chemokines, and extracellular vesicles, have not been fully identified or characterized. Moreover, the interactions between stem cells and other lung cells, such as epithelial cells, fibroblasts, macrophages, and lymphocytes, are not well elucidated. Therefore, more studies are needed to investigate the mechanisms of action of stem cell therapy for PF, to elucidate the molecular pathways involved and identify potential biomarkers for monitoring the treatment response.

### Gathering of long-term safety and efficacy data

The long-term treatment benefits and safety profile of stem cell therapy for PF remain unknown. Most clinical trials have used short follow-up periods (shorter than 12 months) and small sample sizes, as discussed above. Therefore, more rigorous clinical trials with sufficiently long follow-up periods are needed to understand the long-term benefits and risks of stem cell therapy for PF. Potential risks, such as infection, bleeding, thrombosis, immunological reactions, ectopic tissue formation, or malignancy, should also be carefully monitored. Potential benefits, such as improvement in pulmonary function tests (PFTs), quality of life, exercise capacity (6MWD), partial pressure of oxygen in arterial blood (PaO_2_), and survival rate, should be assessed with standardized outcome measures.

These strategies would help optimize stem cell therapy for the treatment of PF and support evidence-based guidance for its widespread use.

## Challenges and limitations

Challenges and limitations in stem cell therapy for PF include the following.

### High treatment cost

Stem cell therapy is expensive and requires specialized equipment, facilities and personnel to produce, store, and deliver stem cells. The cost varies according to the type, source, dose, and delivery method used for administration. Costs may limit treatment accessibility and affordability for many patients with PF with limited financial resources or insurance coverage.^35^

### Need for specialized medical facilities and personnel

Stem cell therapy requires specialized medical facilities and personnel to both extract and administer stem cells. The production, storage, and delivery of stem cells must be strictly monitored to avoid any adverse effects to patients. Moreover, stem cells must be administered by trained staff who can monitor patient condition and manage potential complications. These requirements pose a major challenge to the scalability of therapy.

### Potential risks of immunosuppression

Stem cell therapy may induce immunosuppression in patients with PF, owing to the immunomodulatory properties of stem cells. Although immunosuppression may be beneficial for decreasing inflammation and fibrosis in PF, it may also increase the risk of infection or malignancy in patients with PF who already have compromised immune systems.^36^ Therefore, careful monitoring and prophylaxis are necessary, to prevent or treat any opportunistic infections or cancers that may arise after stem cell therapy.

### Ethical considerations in the use of stem cells derived from embryos

ESCs, pluripotent cells derived from blastocysts, have great potential for transformation into many types of cells and tissues, including lung epithelial and mesenchymal cells. However, the use of ESCs raises ethical concerns regarding the source, status, and rights of human embryos. Some people may consider human embryos to be living beings that deserve respect and protection, whereas others may view human embryos as potential sources of valuable biological materials that can be used for research or therapeutic purposes. These ethical considerations may limit the acceptance and application of ESCs in PF treatment among different stakeholders.^37^

## Conclusions

PF is a serious and progressive lung disease that affects hundreds of thousands of people globally and has a poor prognosis. Currently established treatments have limited scope for actual reversal of the disease process, and improvement in patient quality of life. Thus, to target the mechanisms of tissue damage in PF, newer approaches to treating the disease must be identified.

One such option, stem cell therapy, has shown potential in preclinical studies. Stem cells are pluripotent cells that can alter processes leading to inflammation and fibrosis; repair lung tissue; and restore organ function. Stem cells can be extracted from various sources, such as endogenous lung stem cells, embryonic stem cells, iPSCs, and MSCs, and subsequently delivered for therapeutic purposes through different methods, such as intravenous injection, intratracheal instillation, or inhalation.

However, before the use of stem cells to treat PF, many questions must be answered, and many limitations must be addressed. These include exploring different cell sources, refining delivery methods and dosing regimens, investigating the underlying mechanisms of action, gathering long-term safety and efficacy data, decreasing the high cost of treatment, and addressing ethical issues.

Stem cell therapy can be made more feasible and practical through the provision of evidence-based guidance for its use, before which it cannot serve as the intervention of choice. The various aspects of this therapy, including combination therapies, novel stem cell sources, and advanced tissue engineering, should be further explored. This treatment may have substantial implications for established practice by improving patient outcomes, and quality of life, and potentially enabling individualized treatment approaches. Through deeper understanding of stem cell biology, treatments for PF and many other currently incurable diseases may advance to new levels.

## Source of funding

This research did not receive any specific grant from funding agencies in the public, commercial, or not-for-profit sectors.

## Conflict of interest

The authors have no conflict of interest to declare.

## Ethical approval

Not applicable.

## Consent

Not applicable.

## Author contributions

Concept: MI. Design: MI, MU. Supervision: MI. Data collection and/or processing: MU, MI, SI, MH. Analysis and/or interpretation: MI, MU. Literature search: MU, MI, SI, MH. Writing manuscript: MI, MU, SI. Critical review: MU, MI, MH, SI. Proofreading: MH, SI. All authors have critically reviewed and approved the final draft and are responsible for the content and similarity index of the manuscript.

## Institutional review board statement

Not applicable.

## Declaration of generative AI in scientific writing

During the preparation of this work, the authors used ChatGPT 3.5 to improve the overall language and check grammatical errors after writing the manuscript. The authors then reviewed and edited the content as needed, and take full responsibility for the content of the publication.
